# Principles of diagnosis and management of traumatic pneumothorax

**DOI:** 10.4103/0974-2700.41789

**Published:** 2008

**Authors:** Anita Sharma, Parul Jindal

**Affiliations:** 1 Departments of Postgraduate Medicine and Anaesthesiology, Himalayan Institute of Medical Sciences, Dehradun, Uttarakhand, India

**Keywords:** Diagnosis and management, pneumothorax, trauma

## Abstract

Presence of air and fluid with in the chest might have been documented as early as Fifth Century B.C. by a physician in ancient Greece, who practiced the so-called Hippocratic succession of the chest. This is due to a development of communication between intrapulmonary air space and pleural space, or through the chest wall between the atmosphere and pleural space. Air enters the pleural space until the pressure gradient is eliminated or the communication is closed. Increasing incidence of road traffic accidents, increasing awareness of healthcare leading to more advanced diagnostic procedures, and increasing number of admissions in intensive care units are responsible for traumatic (noniatrogenic and iatrogenic) pneumothorax. Clinical spectrum of pneumothorax varies from asymptomatic patient to life-threatening situations. Diagnosis is usually made by clinical examination. Simple erect chest radiograph is sufficient though; many investigations are useful in accessing the future line of action. However, in certain life-threatening conditions obtaining imaging studies can causes an unnecessary and potential lethal delay in treatment.

Trauma kills approximately 150,000 people each year and is a primary public health concern.[1] Motor vehicle accidents are the most common cause of severe injury and the World Health Organization estimates that by 2020 vehicular injury will be the second most common cause of mortality and morbidity worldwide. According to the most recent data, more than 10% of traumas and accidents terminate in a lethal outcome or a heavy degree of physical inability.[2] Thoracic trauma accounts for one-quarter of trauma deaths, and two-thirds of these deaths occur after the patient reaches hospital.[3] The main problem is collection of air in the pleural cavity causing shift of the mediastinum leading to life-threatening emergency. Promptly recognizing this condition saves lives, both outside the hospital and in a modern intensive care unit (ICU). Because this condition occurs infrequently and has potentially devastating effects, a high index of suspicion and knowledge of basic emergency thoracic decompression are important for all healthcare personnel.

## DEFINITION

A pneumothorax is defined as the presence of air between parietal and visceral pleural cavity.[4] Tension pneumothorax is the accumulation of air under pressure in the pleural space. This condition develops when injured tissue forms a 1-way valve, allowing air to enter the pleural space and preventing the air from escaping naturally. This condition rapidly progresses to respiratory insufficiency, cardiovascular collapse, and ultimately death if, unrecognized and untreated. Favorable patient outcomes require urgent diagnosis and immediate management.

## HISTORY

Physicians defined pneumothorax during the reign of Alexander the Great. Many of the early references to pneumothorax may have been tension pneumothorax, which can be significantly more dramatic in its clinical presentation. The term “pneumothorax” was first coined by a French physician Itard, a student of Laennec in 1803.[5] Needle decompression of the chest for presumed tension pneumothorax has been in practice for many years, but little data exists in the medical literature showing the efficacy of the procedure or reviewing the field-use and incidence of the procedure.

## INCIDENCE

The actual incidence outside of a hospital setting is impossible to determine. In a large study in Israel, spontaneous pneumothoraces occurred in 723 (60.3%) of 1199 cases; of these, 218 were primary and 505 were secondary. Traumatic pneumothorax occurred in 403 (33.6%) patients, 73 (18.1%) of whom had iatrogenic pneumothorax.[6]

In a recent study, 12% of patients with asymptomatic chest stab wounds had a delayed pneumothorax or hemothorax.[3]

## CLASSIFICATION AND TERMINOLOGY OF THE PNEUMOTHORAX

It is usually classified on the basis of its causes. Pneumothoraces are classified as traumatic and nontraumatic (spontaneous).[7] Nontraumatic pneumothoraces are further subdivided into primary (occurring in persons with no known history of lung disease) and secondary (occurring in persons with a known history of lung disease, such as chronic obstructive pulmonary disease).[8]

Pneumothoraces may also be further described as simple pneumothorax (no shift of the heart or mediastinal structures) or tension pneumothorax. It can also be classified as open (“sucking” chest wound) and closed (intact thoracic cage).[7]

## PATHOPHYSIOLOGY OF PNEUMOTHORAX

In normal people, the pressure in pleural space is negative with respect to the alveolar pressure during the entire respiratory cycle. The pressure gradient between the alveoli and pleural space, the transpulmonary pressure is the result of the inherent elastic recoil of the lung. During spontaneous breathing the pleural pressure is also negative with respect to atmospheric pressure.

When communication develops between an alveolus or other intrapulmonary air space and the pleural space, air flows from the alveolus into the pleural space until there is no longer a pressure difference or until the communication is sealed.[9]

### Tension pneumothorax

Tension pneumothorax develops when a disruption involves the visceral pleura, parietal pleura, or the tracheobronchial tree. The disruption occurs when a one-way valve forms, allowing air inflow into the pleural space, and prohibiting air outflow. The volume of this nonabsorbable intrapleural air increases with each inspiration. As a result, pressure rises within the affected hemithorax; ipsilateral lung collapses and causes hypoxia. Further pressure causes the mediastinum shift toward the contralateral side and compresses both, the contralateral lung and the vasculature entering the right atrium of the heart. This leads to worsening hypoxia and compromised venous return. Researchers still are debating the exact mechanism of cardiovascular collapse but, generally the condition may develop from a combination of mechanical and hypoxic effects. The mechanical effects manifest as compression of the superior and inferior vena cava because the mediastinum deviates and the intrathoracic pressure increases. Hypoxia leads to increased pulmonary vascular resistance via vasoconstriction. If untreated, the hypoxemia, metabolic acidosis, and decreased cardiac output lead to cardiac arrest and death.[9,10]

### Traumatic pneumothorax

A traumatic pneumothorax can result from either penetrating or nonpenetrating chest trauma. With penetrating chest trauma, the wound allows air to enter the pleural space directly through the chest wall or through the visceral pleura from the tracheobronchial tree. With non penetrating trauma, a pneumothorax may develop if the visceral pleura is lacerated secondary to a rib fracture, dislocation. Sudden chest compression abruptly increases the alveolar pressure, which may cause alveolar rupture. Once the alveolus is ruptured, air enters the interstitial space and dissects toward either the visceral pleura or the mediastinum. A pneumothorax develops when either the visceral or the mediastinal pleura ruptures, allowing air to enter the pleural space.[11]

## MECHANISM OF INJURY

### Traumatic

**(a) Penetrating trauma** (e.g., stab wounds, gunshot wounds, and impalement on a foreign body) primarily injure the peripheral lung, producing both a hemothorax and pneumothorax in more than 80% of all penetrating chest wounds.

**(b) Blunt trauma** can lead to rib fracture, causes increased intrathoracic pressure and bronchial rupture. Manifested either by “Fallen lung sign” (ptotic lung sign), hilum of lung is below expected level within chest cavity or persistent pneumothorax with functioning chest tube.

### Pulmonary barotraumas

Since the volume of given mass of gas at a constant temperature is inversely proportional to its pressure, so a given volume of air saturated at body temperature expand to 1.5 times the volume at sea level, if it is placed at an altitude of 3050 m, the trapped air in the pleural bleb may rupture resulting in a pneumothorax as seen in air crew members.[12] Similarly in scuba divers, compressed air is delivered to lung by a demand regulators and during ascent barotraumas may occur as ambient pressure falls rapidly, gas contain in the lung expand and cause pneumothorax.[13]

### Iatrogenic pneumothorax

It depends on the circumstances in which it develops [Table 1].

**Table 1:** Causes of iatrogenic pneumothorax according to frequency[11]

Transthoracic needle aspiration or biopsy	24%
Subclavian or jugular vein catherterization	22%
Thoracentesis	20%
Closed pleural biopsy	8%
Mechanical ventialtion	7%
Cardiopulmonary resuscitaion	
Nasogastric tube placement	
Transbronchial biopsy	
Tracheostomy	
Liver biopsy	
Miscellanseous:
Markedly displaced thoracic spine fracture	
Acupuncture has been reported to result in pneumothorax in recent years	
Colonoscopy and gastroscopy have been implicated in case reports	
Intravenous drug abusers if they choose neck veins	

The leading cause of iatrogenic pneumothorax is transthoracic needle aspiration. Two factors may be responsible for it, depth and size of the lesion. If the lesion is deeper and the size is smaller chances of traumatic pneumothorax increases.

The second leading cause of iatrogenic pneumothorax is central cannulation, due to the increasing number of patients requiring intensive care.

Inadvertent subclavian arterial puncture is a relatively common complication of subclavian venepuncture.[14] The overall reported incidence is in the range of 1-13% with 2-5% being typical. This incidence increases to about 40% if multiple attempts are made. Thoracentesis is probably the third leading cause of iatrogenic pneumothorax .This can be reduced if it is done under ultrasound guidance. In a study analyzing outcomes of 418 invasive procedures, the incidence of iatrogenic pneumothorax was 13% for computed tomography (CT)-guided transthoracic fine needle aspiration (TFNA), 7.1% for pleural biopsy, 16.6% for transbronchial biopsy, 7.1% for fluoroscopy guided TFNA, and 1.5% for thoracentesis.[15] Mechanical ventilation causing pneumothorax has come down because with newer ventilatory mode it is possible to ventilate patients with lower peak pressures and lower mean airway pressure. Other procedures which may be responsible are, transpleural and transbronchial lung biopsies, cardiopulmonary resuscitation, thoracic acupuncture,[16] and in intravenous drug abuser using neck veins.

## DIAGNOSIS

Diagnosis of pneumothorax is done by thorough clinical examination and investigations. However, clinical interpretation of the presenting signs and symptoms is crucial for correctly diagnosing and treating the condition.

### Common early findings include[18–22] [Table 2]

**Table 2:** Classic signs of pneumothorax[17]

Trachea	→
Expansion	↓
Percussion note	↑
Breath sounds	↓
Neck veins	↑

Chest pain

Dyspnea

Anxiety

Tachypnea

Tachycardia

Hyper resonance of the chest wall on the affected side

Diminished breath sounds on the affected side

### Whereas late findings includes

Decreased level of consciousness

Tracheal deviation toward the contralateral side

Hypotension

Distension of neck veins (may not be present if hypotension is severe)

Cyanosis

## IMAGING STUDIES

### Chest radiography

**Figure 1:**
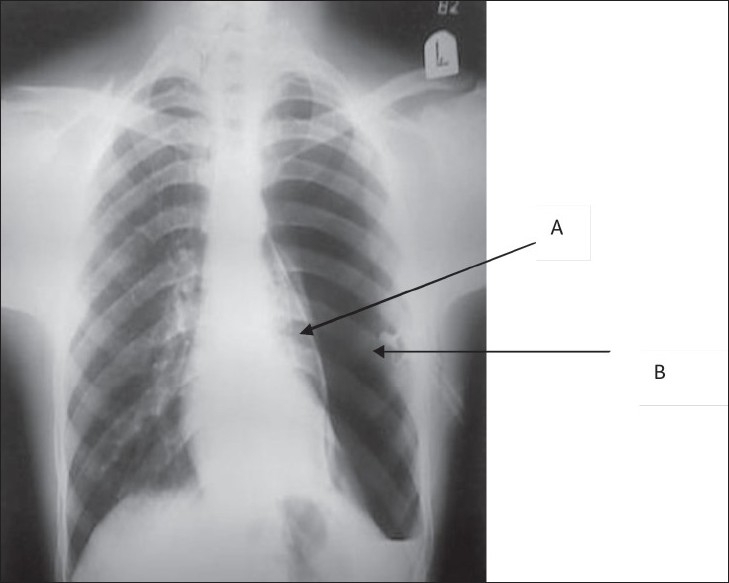
Chest X-Ray showing pneumothorax secondary to blocked chest tube. A. Pleural white line B. Blocked chest tube

**Figure 2:**
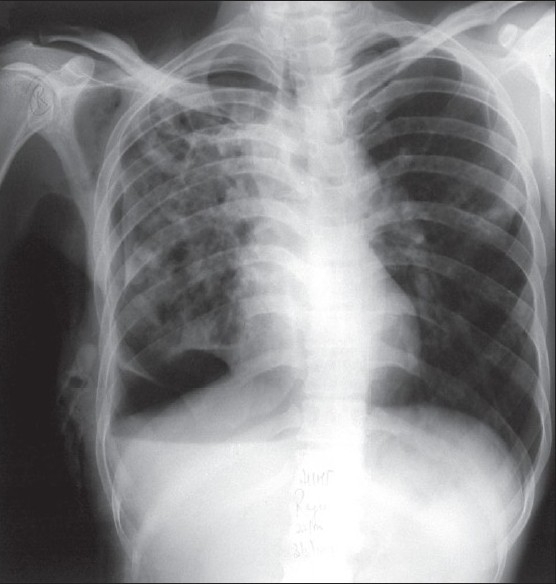
Depressed right hemidiaphragm due to pneumothorax

**Figure 3:**
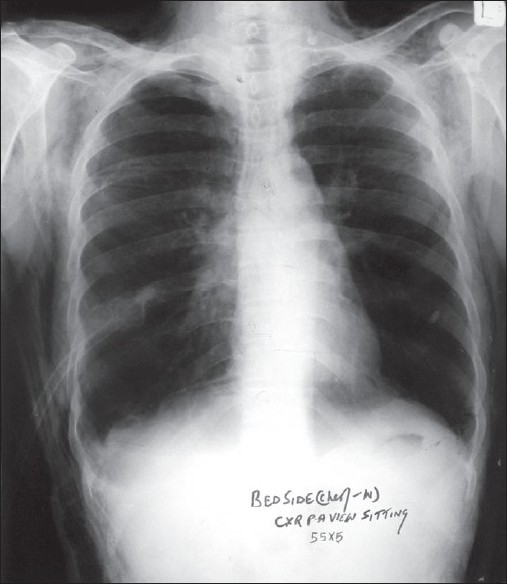
Subcutaneous emphysema

It is diagnostic in majority of the cases and findings are classical [Figures 1–3].

In some patients, it may be preferable to radiologically confirm and localize tension pneumothorax before subjecting the patient to potential morbidities arising from decompression. However, this consideration should be limited to patients who are awake, stable, not in advanced stages of tension and when an immediate chest film can be obtained, with facilities to perform urgent decompression if needed.

Serial chest radiographs every 6hrs on the first day after injury to rule out pneumothorax is ideal, but two or three chest X-ray taken every 4-6hrs are sufficient.

Air in the pleural cavity, with contralateral deviation of mediastinal structures, is suggestive of a tension pneumothorax. Chest radiographic findings may include increased thoracic volume, increased rib separation, ipsilateral flattening of heart border, contralateral mediastinal deviation, and the midiaphragmatic depression.

Rotation can obscure a pneumothorax and mimic a mediastinal shift.

In evaluating the chest radiograph, first impressions of pneumothorax size can be misleading. To assist in determining the size of pneumothorax on the radiograph, a 2.5-cm margin of gas peripheral to the collapsing lung corresponds to a pneumothorax of about 30%. Complete collapse of the lung is a 100% pneumothorax.

Supine chest AP films are notoriously inaccurate. Because they result in air spreading out over the anterior chest, supine films often appear normal, even in the presence of significant air. Frequently, the only indication is the “deep sulcus sign,”[19] so named because of the appearance of an especially deep costovertebral sulcus.

In rare circumstances when there is bilateral pneumothorax patient may appear in severe respiratory distress with engorged neck vein, one may not find signs of mediastinal shift and findings on both sides of the lung will also be same [Tables 3 and 4].

**Table 3:** Radiological findings

Visceral pleural white line	Convexity towards hilum
Absence of lung markings	Distal or peripheral to the visceral pleural white line
Displacement of mediastinum	Towards opposite side
Deep sulcus sign[19]	On frontal view, larger lateral costodiaphargmatic recess than on opposite side
	Diaphragm may be inverted on side with deep sulcus
Total/subtotal lung collapse	This is passive or compressive atelectasis
Radiographic signs in upright position	Sharp delineation of visceral pelural by dense pleural space Mediastinal shift to opposite side Air-fluid level in pleural space on erect chest radiograph White margin of visceral pleura separated from parietal pleura Usually seen in the apex of the lung Absence of vascular markings beyond visceral pleural margin May be accentuated by an expiratory film in which lung volume is reduced while amount of air in pneumothorax remains constrants so that relative size of pneumothorax appears to increase
Radiographic signs in supine position (difficult to see)	Anteromedial pneumothorax (earliest location) Outline of medial diaphragm under cardiac silhouette Deep sulcus sign

**Table 4:** Pitfalls in the diagnosis of pneumothorax with chest X-ray

Skin fold	Thicker than the thin visceral pleural white line
Air trapped between chest wall and arm	Will be seen as a lucency rather than a visceral pleural white line
Edge of scapula	Follow contour of scapula to make sure it does not project over chest
Overlying sheets	Usually will extend beyond the confines of the lung
Hair braids	-
Emphysematous bullae	Convexity laterally

### Chest CT scanning

A CT scan is more sensitive than a chest radiograph in the evaluation of small pneumothoraces and pneumomediastinum, although the clinical significance of these occult pneumothoraces is unclear, particularly in the stable nonintubated patient.[23]

The occult pneumothorax is being diagnosed more frequently as methods of evaluating and diagnosing trauma patients become more sensitive. At present, CT scan is the gold standard for detecting occult traumatic pneumothorax not apparent on supine chest X-ray radiograph.[24]

### Ultrasonography

Use of bedside ultrasonography in the diagnosis of pneumothorax is a relatively recent development. In some trauma centers, pneumothorax detection is included as part of their focused abdominal sonography for trauma (FAST) examination.[25]

Ultrasonographic features used in the diagnosis of pneumothorax include absence of lung sliding (high sensitivity and specificity), absence of comet-tail artifact (high sensitivity, lower specificity), and presence of lung point (high specificity, lower sensitivity). In a study, ultrasonography performed on patients with blunt thoracic trauma had 94% sensitivity and 100% specificity for pneumothorax detection compared with spiral CT scanning[26,27] [Table 5].

**Table 5:** Conventional ultrasonic signs in the lung

Findings	Description
Pleural line	Horizontal hyper-echoic line between upper and lower ribs, identified by acoustic shadows
Lung-sliding	Forward-and-back movement of visceral pleura against parietal pleura in real-time motion
Comet-tail artifacts	Are hyper-echoic reverberation artifacts arising from the pleural line, laser-beam-like and spreading up to the edge of the screen

### Arterial blood gas analysis

Arterial blood gas (ABG) does not replace physical diagnosis nor should treatment be delayed while awaiting results if symptomatic pneumothorax is suspected. However, ABG analysis may be useful in evaluating hypoxia, hypercarbia, and respiratory acidosis.

### Electrocardiography

In left-sided pneumothorax electrocardiogram (ECG) shows: rightward shift of the frontal QRS axis, diminution of the precordial R voltage, decrease in QRS amplitude, and precordial T-wave inversion. With right pneumothorax ECG may show diminution of the precordial QRS voltage, right axis deviation, and a prominent R wave in V2 with associated loss of S wave voltage, mimicking posterior myocardial infarction. All these changes are thought to be due to mechanical effects and should not be taken for cardiac ischemia or infarction.

## CLINICAL DIAGNOSIS OF AN IATROGENIC PNEUMOTHORAX

The diagnosis of an iatrogenic pneumothorax should be suspected in any patient treated by mechanical ventilation whose clinical condition suddenly deteriorates[4] [Table 6].

**Table 6:** Occurrence of pneumothorax in a mechanically ventilated patient

Finding	Cause
Sudden onset of tachycardia, hypotension	Tension pneumothorax impending venus return
Increase in peak airway pressure	External lung compression
Sudden decline in oxygen saturation	Lung collapse
Distressed patient	To fight ventilator

The diagnosis should be suspected in any patient who become more dyspneic after a medical or a surgical procedure that is known to be associated with the development of the pneumothorax. However, chest X-ray immediately after central canulation may not show pneumothorax.

## TREATMENT

Management depends not only on the clinical setting, the site where we treat the patient (site of trauma or in the hospital), any procedure which is causing pneumothorax, but also on the size of pneumothorax, associated co-morbid condition, whether it is open/closed and simple/tension pneumothorax.

Method to estimate the correct size of pneumothorax are controversial. There are currently two methods described in adults, if the lateral edge of the lung is >2cm from the thoracic cage. Then, this implies pneumothorax is at least 50% and hence large in size. Calculate the ratio of transverse radius of the pneumothorax (cubed) to the transverse radius of hemithorax (cubed). To express the size as a percentage, multiply the fraction size by 100.[28,29]

### First aid

Airway, breathing, and circulation should be checked in all the patients of chest trauma. Patency of the airway and the adequacy of the ventilatory efforts should be evaluated with the assessment of the integrity of the chest and the circulatory status as pericardial tamponade can also cause signs and symptoms similar to tension pneumothorax. Upright positioning may be beneficial if there is no contraindication to it like spinal injury.

Penetrating wounds (also known as ‘sucking chest wounds’) require immediate coverage with an occlusive or pressure bandage made air-tight with clean plastic sheeting. The sterile inside of a plastic bandage packaging can be used in an emergency situation. No patient with penetrating chest wound should be left unattended as tension pneumothorax or other immediately life-threatening respiratory emergency can arise.

A thin needle can be used for this purpose, to relieve the pressure and allow the lung to reinflate in suspected tension pneumothorax. An untreated pneumothorax is an absolute contraindication for evacuation or transportation by flight.

Hemothorax can be associated with pneumothorax, and the patient may require immediate intravenous infusion hence large-bore iv canula should be placed.

### Oxygen therapy

Immediately administer 100% oxygen. Administration of supplemental oxygen accelerates the rate of pleural air absorption in clinical and experimental situations. By breathing 100% oxygen instead of air, alvelolar pressure of nitrogen falls, and nitrogen is gradually washed out of tissue and oxygen is taken up by vascular system. This causes substantial gradient between tissue capillary and the pneumothorax space, this results in multifold increase in absorption from pleural space. It is recommended that hospitalized patient with any type of pneumothorax who is not subjected to aspiration or tube thoracostomy should be treated with supplemental oxygen at high concentration. Normally 1.25% of the volume of is absorbed in 24 h, hence 10% of the volume is absorbed in 8 days and 20% would be in 16 days and so on.[30]

Majority of the patients with small pneumothoraces often are managed with oxygen administration no treatment other than repeat observation via chest X-rays may be required.

Several prospective studies in both emergency medicine and surgery literature dating back to the mid-1980s have supported the use of needle aspiration and/or small-bore catheter placement for the treatment of pneumothoraces.[31–34]

Complications of tube thoracostomy include death, injury to lung or mediastinum, hemorrhage (usually from intercostal artery injury), neurovascular bundle injury, infection, bronchopleural fistula, and subcutaneous or intraperitoneal tube placement.

### Simple aspiration

It is done by a plastic iv canula instead of traditionally used needle which was associated with risk of laceration of lung. The site of second intercostals space in midclavicular line is conventional. It can also be performed in fifth intercostals space in anterior axillary line to prevent life threatening hemorrhage. Available literature of American College of Chest Physician (ACCP) and British Thoracic Society (BTS) says that the needle aspiration and/or small catheter insertion are effective, comfortable, safe, and economical alternatives to thorcostmy in selected patients.[35,36]

### Tube thoracostomy

This procedure is recommended if simple aspiration proves ineffective and thoracoscopy is not readily available. The site for the insertion is same as for simple aspiration. It rapidly results in the re-expansion of the underlying lung and does not require prolonged hospitalization. Risk of re-expansion pulmonary edema is greater when the lung is re-expanded rapidly, it is probably better to use water seal and to avoid suction for the first 24 h of tube thoracostomy. Now-a-days Malecot's catheters are replaced by pre-packed disposable plastic tube with the long central metal trocar (18-24 Fr Gauge). Correct placement of the tube is seen as the stream of the bubbles during expiration and coughing and the rise on the level of fluid in the under water seal during inspiration.

If the lung remains unexpanded or if there is a persistent air leak 72 h after tube thoracostomy, consideration should be given to performing thoracoscopy or thoracotomy.[37–39]

### Important points to remember

- As tension pneumothorax is a life-threatening condition, the diagnosis of a tension pneumothorax should be made based on the history and physical examination findings. A chest radiograph or CT scan should be used only in those instances where one is in doubt regarding the diagnosis and when the patient's clinical condition is sufficiently stable.
- Premature diagnosis of tension pneumothorax in a patient without respiratory distress, hypoxia, hypotension, or cardiopulmonary compromise should not be made. Immediate portable chest X-ray must be done to confirm the diagnosis.
- Consider the diagnosis of a pneumothorax and/or tension pneumothorax with blunt and penetrating trauma. In the patient with blunt trauma; mental status changes, hypoxia and acidosis may be attributed to a suspected intracerebral injury rather than a tension pneumothorax. Portable chest radiography should always be included in the initial radiographic evaluation of major trauma.
- Myocardial rupture with tamponade may clinically mimic tension pneumothorax.
- Maintain a high index of suspicion for a tension pneumothorax in patients using ventilators who have a rapid onset of hemodynamic instability or cardiac arrest, particularly if they require increasing peak inspiratory pressures.
- Avoid assuming that a patient with a chest tube does not have a tension pneumothorax if he or she has respiratory or hemodynamic instability. Chest tubes can become plugged or malpositioned and cease to function.
- Avoid the “one size fits all” approach for tube thoracostomy placement.
- Tube thoracostomy is an extremely painful procedure. In stable patients, adequate analgesia/sedation should be administered, followed by generous amounts of local anesthetics when chest tubes are placed.
- An initial parenteral dose of a first-generation cephalosporin should be administered for chest tube insertion in the emergency department to decrease the risk of empyema and pneumonia.
- Small pneumothoraces should be treated with thoracostomy tubes if the patient is undergoing mechanical ventilation or undergoing air transport prior to transfer to another facility.

## PREVENTION

- Advise persons to wear safety belts and passive restraint devices while driving.
- When subclavian vein cannulation is required, use the supraclavicular approach rather than the infraclavicular approach when possible to help decrease the likelihood of pneumothorax formation.
- Transbronchial, transthoracic, and other procedures preferably be done under ultrasound guidance.

## CONCLUSION

Pneumothorax has been recognized condition since ancient times. Various methods for the diagnosis and treatment are advised from time to time. Traditional approaches to the diagnosis and management of pneumothorax are being challenged, and physicians should keep an open mind regarding new approaches to this condition. As CT scans have become cheaper and more widely utilized their role in diagnosing pneumothorax is also evolving and being more clearly defined. More cases of small pneumothorax are being diagnosed, but management decisions are not necessarily being altered. Less costly and less painful alternatives (other than standard tube thoracostomy and admission) exist for many etiologies, and more patients are being discharged home than in the past. Understanding these trends is critical to providing optimal care for patients with pneumothorax.
